# Annotations

**Published:** 1904-11-05

**Authors:** 


### Annotations.

### The Work of the Industrial Schools.

Students of social progress must needs include in their survey of affairs the history of the London School Board. Aid in this direction will be afforded by the Yellow Books which have been planned as an official record of the various activities of the Board. One of these, dealing with the work of the industrial schools, has just been issued, and it is certainly a most interesting and encouraging volume. Some notion of the problem which presented itself when the School Board came into existence can be formed from the statement that in 1851 it was estimated that there were in London not less than 30,000 neglected and degraded children. Between that date and 1870 some attempt was made to deal with this class, but no considerable measure of success was attained. Even the School Board, when first created, was not able to give much attention to the question, in view of the many other claims and duties which demanded immediate consideration. The measure of progress is obtained by contrasting the "distinctly inferior" work then with the most recent verdict, which pronounces that "the work in an industrial school is probably as good as the average Board school." When it is considered that the children brought under the influence of these schools come from surroundings which almost inevitably breed criminals, that they are developed into sailors, soldiers, domestic servants, etc., and that the percentage of them who fail in after life is "extremely small," it is obvious that the industrial schools are playing no small part in the development of a social revolution.

### A Great Sanitarian.

The cause of sanitary science, and the adjustment of its principles to the demands of practical life, have lost a most influential and devoted leader in the death of Dr. J. B. Russell. Of recent years the vigour and force of his personality have to some extent been hidden from public attention under the official guise of the Local Government Board for Scotland. Thus it is more than probable that his services in the capacity of medical adviser to this branch of the executive will be less remembered than the great work he accomplished as medical officer of health for Glasgow. To this position he succeeded in 1872, and he continued in office for the long period of 26 years. It is not too much to say that during that time the value of his services to the local community cannot be expressed. The conception of the public health as a matter concerning the general welfare, and demanding official guidance and control, hardly existed in the public conscience in the early seventies, and suggestions of inspection and regulation were suspected as invasions of the rights of the individual. To have carried public opinion from this point to the present attitude of confidence and trust in the sanitary organisation and its several departments means not only a great achievement, but a skill in method which is little short of genius. This, indeed, was one of the secrets of Dr. Russell's success. He sought great ends and had large conceptions of public polity, and at the same time he recognised that to obtain these ambitions it was essential to educate rather than to compel the community. From the sanitary horrors of the Glasgow of 1872 to the ordered scheme of public health of 1898 is a wide contrast, and the progress which it means was in the main due to the steady devotion and patience of Dr. J. B. Russell. Even those who knew neither the man nor the local work which he accomplished may readily appreciate his capacity and skill by a perusal of his official reports and papers. In these plainly appear both the sanitary reformer and the practical statesman, in association, too, with the literary artist. It is much to be hoped that an effort will be made to edit a selection of these papers, not only as a memorial of a man who served well his day and generation, but also as a source of instruction in the problems of sanitary science as these are presented by a great and growing city.

### The Russian Red Cross Society.

Such damaging statements have been made in reference to the administration of the funds of the Russian Red Cross Society that an official announcement had become essential, both in justice to the responsible authorities and also in order to prevent the receipts of the Society from suffering. One assertion is to the effect that throughout the Muscovite Empire public and private associations have, quite independently of the Russian Red Cross Society, sent out ambulance trains to the seat of the war, simply because they have lost all faith in the honesty of the officials. They have only, it is said, too substantial grounds for withdrawing their confidence, when they know that persons who have been dismissed for embezzlement in other positions are entrusted with the administration of funds for the benefit of suffering soldiers at the front. An individual of this class is described as having been charged with the task of expending 600,000 roubles without spending a single penny of the £60,000 upon the object for which it was intended. It is added that the only punishment the delinquent received was his recall, and that on his return he was given another appointment at the Red Cross Society's headquarters at St. Petersburg. It is also alleged that an association of nobles in the south of Russia wished to equip a special ambulance, and having completed their preparations, endeavoured to obtain official permission. This, it is averred, was so inexplicably delayed that a member of the Association went to St. Petersburg to make personal inquiries. As the result of much persistence, he ascertained that the obstacle was a highly-placed official who would not give the necessary sanction until he had received a substantial douceur. The report is that it was considered advisable to propitiate him, and the ambulance was then allowed to proceed. The official reply to these allegations appears to be that they are part of a malicious campaign against the Russian Red Cross Society, set on foot deliberately for the purpose of diminishing the contributions to the fund for the Russian wounded at the front. It is admitted that there was a case of malversation, but that it took place before the war, although it was not discovered until after the outbreak of hostilities. This admission seems to us to be a reason for a more searching inquiry than has apparently yet been instituted. At any cost to individual reputations the honoured name of the Red Cross should be saved from obloquy.

